# Exploring the biosynthetic gene clusters in *Brevibacterium*: a comparative genomic analysis of diversity and distribution

**DOI:** 10.1186/s12864-023-09694-7

**Published:** 2023-10-19

**Authors:** Andrés Cumsille, Néstor Serna-Cardona, Valentina González, Fernanda Claverías, Agustina Undabarrena, Vania Molina, Francisco Salvà-Serra, Edward R.B. Moore, Beatriz Cámara

**Affiliations:** 1 Centro de Biotecnología DAL, Universidad Técnica Federico Santa María, Valparaíso, Chile; 2 Department of Infectious Diseases, Institute for Biomedicine, Sahlgrenska Academy, University of Gothenburg, Gothenburg, Sweden; 3 Department of Clinical Microbiology, Region Västra Götaland and Sahlgrenska Academy, Culture Collection University of Gothenburg (CCUG), Sahlgrenska University Hospital, University of Gothenburg, Gothenburg, Sweden; 4 Centre for Antibiotic Resistance Research (CARe), University of Gothenburg, Gothenburg, Sweden; 5 Microbiology, Department of Biology, University of the Balearic Islands, Palma de Mallorca, Spain

**Keywords:** Biosynthetic gene clusters, Comparative genomics, *Brevibacterium*

## Abstract

Exploring *Brevibacterium* strains from various ecosystems may lead to the discovery of new antibiotic-producing strains. *Brevibacterium* sp. H-BE7, a strain isolated from marine sediments from Northern Patagonia, Chile, had its genome sequenced to study the biosynthetic potential to produce novel natural products within the *Brevibacterium* genus. The genome sequences of 98 *Brevibacterium* strains, including strain H-BE7, were selected for a genomic analysis. A phylogenomic cladogram was generated, which divided the *Brevibacterium* strains into four major clades. A total of 25 strains are potentially unique new species according to Average Nucleotide Identity (ANIb) values. These strains were isolated from various environments, emphasizing the importance of exploring diverse ecosystems to discover the full diversity of *Brevibacterium*. Pangenome analysis of *Brevibacterium* strains revealed that only 2.5% of gene clusters are included within the core genome, and most gene clusters occur either as singletons or as cloud genes present in less than ten strains. *Brevibacterium* strains from various phylogenomic clades exhibit diverse BGCs. Specific groups of BGCs show clade-specific distribution patterns, such as siderophore BGCs and carotenoid-related BGCs. A group of clade IV-A *Brevibacterium* strains possess a clade-specific Polyketide synthase (PKS) BGCs that connects with phenazine-related BGCs. Within the PKS BGC, five genes, including the biosynthetic PKS gene, participate in the mevalonate pathway and exhibit similarities with the phenazine A BGC. However, additional core biosynthetic phenazine genes were exclusively discovered in nine *Brevibacterium* strains, primarily isolated from cheese. Evaluating the antibacterial activity of strain H-BE7, it exhibited antimicrobial activity against *Salmonella enterica* and *Listeria monocytogenes*. Chemical dereplication identified bioactive compounds, such as 1-methoxyphenazine in the crude extracts of strain H-BE7, which could be responsible of the observed antibacterial activity. While strain H-BE7 lacks the core phenazine biosynthetic genes, it produces 1-methoxyphenazine, indicating the presence of an unknown biosynthetic pathway for this compound. This suggests the existence of alternative biosynthetic pathways or promiscuous enzymes within H-BE7’s genome.

**Supplementary Information:**

The online version contains supplementary material available at 10.1186/s12864-023-09694-7.

## Background

The decrease in the discovery rate of new drugs to cope with the spread of antimicrobial-resistant bacteria is a worldwide public health issue [1]. Traditionally, antimicrobial discovery relies on the isolation of natural products (NP) from cultured microbes, where bacterial products comprise more than half of the known natural antimicrobials and antivirals [2]. Nevertheless, several advances have opened new ways to overcome this threat, such as informatic-based strategies, such as genome mining [3, 4]. Altogether, thanks to massive sequencing techniques upraise, there have been major identification of Biosynthetic Gene Clusters (BGCs) responsible for producing antimicrobial compounds [5, 6], and the search for these gene clusters has become an important means for revealing the biotechnological potential of different bacterial species [7, 8].

Bacteria belonging to the phylum Actinomycetota represent the most prominent group of microorganisms for the production of bioactive compounds [9, 10], accounting for the production of more than 64% of natural product antibiotic classes [11]. Specialized metabolites are often small molecules that are not essential for growth and reproduction, although, they provide advantages for the survival of producer organisms [6]. One example is the production of antimicrobial compounds, which inhibits the growth of surrounding organisms that compete for the same resources [12–14]. These compounds are synthesized by Biosynthetic Gene Clusters (BGCs), which are groups of genes, physically clustered, that encode a biosynthetic pathway to produce a specialized metabolite [15], comprising different classes, such as polyketides, peptides, saccharides, terpenes, and alkaloids [14, 16]. The capabilities of strains of taxa of the Actiomycetota to produce bioactive specialized metabolites rely on their genomic potential and the presence of these BGCs [13, 17–20].

*Brevibacterium* (within the famil*y Brevibacteriaceae*, order Micrococcales, class Actinomycetia, phylum Actinomycetota) are nonmotile, non-spore-forming rod-shaped bacteria [21]. There are 70 described species of *Brevibacterium* [22], isolated from several ecosystems, such as soil [23–25], aquatic [26], human-derived [27, 28] and marine [29–33], but mostly from dairy products such as cheese, where they are responsible for conferring key organoleptic features and pigments [34–38]. *Brevibacterium* are known to produce specialized metabolites, such as Linocin M18 from *B. linens* M18 [39], biosurfactants [40], and also phenazines from *Brevibacterium* sp. KMD 003 [41], and *B. iodinum* ATCC 49,514^T^ [36, 42]. Additionally, a negative correlation has been observed between the presence of *B. aurantiacum* in cheeses and the growth of *Listeria monocytogenes* [43].

Phenazines and their derivatives have been reportedly produced by different genera other than *Brevibacterium*, such as *Pseudomonas, Streptomyces, Kitasatospora, Nocardia*, and *Burkholderia* [42, 44–47]. They exhibit a broad range of biological activities [44]. The BGCs responsible for the biosynthesis of phenazines are reported in *Streptomyces virginiae* DSM 1042 [47], *Streptomyces anulatus* 9663 [48], *Pseudomonas chlororaphis* H18 [46] and *Kitasatospora* sp. HKI 714 [45], among others. The pathways associated with the production of phenazines of *S. anulatus* consist of a BGC, where six coding genes are involved in the biosynthesis of mevalonate, and seven are related to the assembly and biosynthesis of phenazine [48]. Phenazine compounds could also give specific advantages to producers and surrounding strains, increasing their resistance to antibiotics [49].

Previous comparative genomics studies of the genus *Brevibacterium* analysed 23 genomes and discovered several features that could confer adaptive advantages to cheese-related strains, for instance, the production of osmoprotectants such as ectoine, the production of proteases, lipases, and siderophores, as well as the presence of bacteriocin BGCs in most strains [36]. Another study, mainly focused on cheese-related *B. aurantiacum*, assessed their antimicrobial activity against various bacterial species, however, no activity was observed. Also, the authors revealed that most strains of *Brevibacterium* species used for cheese production, belong to *B. aurantiacum* species [34]. Both studies contributed to publishing several genomes of *Brevibacterium* in public databases [34, 36], and uncovered the presence of BGCs for ribosomal synthesized and post-translational modified peptides (RiPPs) in several strains [34, 36]. Altogether, the presence of phenazine-related BGCs has been reported in strain *B. iodinum* ATCC 49514^T^ and in four other cheese-related strains [36].

A comparative genomic study of 98 *Brevibacterium* genomes isolated from several environments was undertaken, using a tested pipeline for genome quality analysis [7]. To gain a deeper understanding of the ecological and phylogenetic factors influencing the presence of BGCs in this genus, we employed prediction and networking tools for BGC analysis. Understanding the function and evolution of specialized metabolites in their natural ecosystems could help inspire and produce more efficient and novel metabolites in the future [50]. Additionally, we report the genome sequence of *Brevibacterium* sp. H-BE7, isolated from marine sediments of the Comau Fjord (Northern Patagonia, Chile) [51]. We evaluated the antibacterial activity of the crude extract against clinically relevant model strains and employed liquid chromatography coupled with high-resolution mass spectrometry (LC-HRMS) to identify and characterize the compounds present in the extract, a process commonly referred to as dereplication.

## Methods

### Genome sequencing

*Brevibacterium* sp. H-BE7 was isolated from marine sediments of the Comau Fjord, in the Huinay Marine Protected area in Southern Chile [51]. Initial identification, determined by 16 S rRNA gene sequence comparative analysis, showed that strain H-BE7 clustered within the *Brevibacterium* genus, closely related to *B. oceani* [51]. To sequence the genome of *Brevibacterium* sp. H-BE7, DNA extraction was achieved, using Wizard Genomic DNA extraction kit (Promega), DNA quality and purity were assessed with Qubit dsDNA BR Assay kit (Thermo Fisher Scientific), using NanoDrop 2000 Spectrophotometer and an 0.8% agarose gel electrophoresis. Genome sequencing was first achieved by Illumina, provided by the Culture Collection of the University of Gothenburg. DNA was used to construct a library, using a TruSeq Nano DNA High Throughput Library Preparation Kit (Illumina, USA) and sequenced using an Illumina MySeq platform (Illumina, USA) to obtain 300 bp paired-end reads (SciLifeLab, Sweden). Subsequently, the isolated DNA was used to build a sequencing library, using an Oxford Nanopore Technologies (ONT) Rapid Sequencing Kit (SQK-RAD004) (Oxford Nanopore Technologies, UK). The prepared library was loaded into an ONT MinION R9.4 Flow Cell (FLO-MIN106) and sequenced in a MinION Mk101B sequencing device for 48 h. Sequences were submitted to the Sequence Read Archive (SRA) under the accession number PRJNA977705.

A hybrid genome assembly was performed, where Illumina raw reads were trimmed using Sickle v0.5 [52] and corrected using SPAdes v3.10.1 [53] error-correction flag. Nanopore raw reads were filtered using NanoFilt 2.0.0 [54] using a Q10 threshold. Nanopore and Illumina reads were assembled using Unicycler v 0.4.4 [55]. The genome of *Brevibacterium* sp. H-BE7 is deposited in GenBank/ENA/DDBJ under GCA_030227105.1 accession. Genome assembly was annotated using Prokka v 1.13 [56].

Circular genome representation was achieved, using GenoVi v0.2.1 [57], a software that performs circular genomic representations using Circos [58]. GenoVi automatically calculates and formats the GC-content and GC-skew, using a modified version of GC-analysis.py and skewIT, respectively [59], graphics each Protein Coding Sequences (CDSs), and calls for DeepNOG [60] to classify them into Clusters of Orthologous Groups of Proteins (COGs). BGCs were predicted, using standalone antiSMASH v6.0.1 [61], with Cluster BLAST option, and displayed into the circular genomic representation, manually editing a Circos configuration file created by GenoVi.

### Phylogenomic analysis

All *Brevibacterium* genomes with less than 200 contigs available on the National Center for Biotechnology Information (NCBI) [62], were downloaded (116 entries as of July 21st, 2021). All downloaded genomes were quality-checked as previously described [7]. Briefly, CheckM v1.1.3 [63] was used, and only genomes with a completeness of > 98% and contamination of < 5% were selected. Additionally, a manual evaluation was accomplished, to discard redundant genomes. After applying all filters, 98 *Brevibacterium* genomes, including strain H-BE7, were included for further analysis (Table S1).

A phylogenomic analysis was performed employing Orthofinder v2.5.4 [64], using *Kocuria rosea* ATCC 186^T^ as an outgroup. DIAMOND aligner [65] was used to retrieve orthogroups, MAFFT [66] for multiple sequence alignment, and FastTree v2.1.3 [67] for cladogram inference. For visualization, iTOL v5 [68] was used.

### Pangenome analysis

For the pangenomic analysis, the anvi’o v7 [69] workflow for microbial pangenomics was followed [70]. Each genome was stored as an anvi’o contigs database, and then a pangenome analysis was computed. Anvi’o pangenome analysis uses DIAMOND, to calculate the similarity between gene calls of each genome, then uses Markov Cluster Algorithm (MCL) to identify clusters or groups of similar genes among selected genomes and finally organize gene clusters, using Euclidean distance. The pangenome was constructed, using the phylogenomic cladogram information, to observe similarities among clades. Additionally, a Blast-based Average Nucleotide Identity (ANIb), using PyANI [71] was assessed, to evaluate the similarity among strains. The ANIb algorithm performs BLAST searches of 1,000 bp genomic fragments against a target genome [72]. Finally, a functional enrichment analysis was performed, using the anvi’o compute functional enrichment program [73]. Annotation of the gene clusters, using the COGs database was performed, and an enrichment score for each function was computed to find out if the occurrence of certain COGs functions is greater in specific niches or clades of *Brevibacterium*.

### Biosynthetic gene cluster (BGC) analysis

To evaluate the presence of BGCs among *Brevibacterium* genus, BGCs were predicted for each genome using antiSMASH v6.0.1. A BGC Network was constructed, using BiG-SCAPE 1.1.2 [74]. Several raw distance cut-offs were tested, ranging from 0.3 to 0.9, with a step of 0.1, where 0.6 was selected. A preliminary network was assessed, using all MIBiG BGCs, those that connected with BGCs from *Brevibacterium* strains were further selected for a final network. Network visualization was achieved using Gephi 0.9.2 [75].

To analyse the phylogenetic relationship between BGCs, CORASON [74] was used. To retrieve BGCs not predicted with antiSMASH, strain H-BE7 PKS and RiPPs BGCs were used as query applying the cblaster tool, to search locally within the 98 *Brevibacterium* genomes as a DIAMOND database [65]. Cblaster uses a query to search against the database and then retrieves the selected BGCs in GenBank format. Clinker [76] was then used to visualize the similarities between BGCs, displaying the similarities bigger than 60% as lines.

The genomes of *Rothia kristinae* were downloaded from NCBI, using the accession number provided by Oliveira et al., 2022. BGCs were predicted, using antiSMASH v6.1.1 and PKS BGCs were compared to strain H-BE7, using Clinker.

### Growth conditions, antimicrobial assays, and chemical dereplication

To evaluate the antibacterial activity of *Brevibacterium* sp. H-BE7, fermentations and ethyl acetate (EtOAc) crude extracts were performed as previously described [51, 77]. Briefly, a 50 mL culture of strain H-BE7 on ISP2 medium (4 g l ^− 1^ dextrose, 10 g l ^− 1^ malt extract and 5 g l ^− 1^ yeast extract) prepared with artificial sea water (ASW) was incubated for 10 days at 30 °C with continuous shaking at 180 rpm. The culture was centrifuged at 5,000 rpm, the pellet discarded, and the supernatant was extracted twice with EtOAc 1:1. The organic phase was then concentrated in a rotary evaporator and completely dried in a speed vacuum. The dried crude extract was stored at -20 °C until further use.

Antimicrobial activity was assessed using crude extract dissolved in dimethyl sulphoxide (DMSO) (10% v/v) to a final concentration of 5 mg mL^− 1^. The model bacteria used for screening are *Staphylococcus aureus* NBRC 100,910^T^; *Listeria monocytogenes* 07PF0776; *Salmonella enterica* subsp *enterica* LT2^T^; *Escherichia coli* FAP1 and *Pseudomonas aeruginosa* DSM 50,071^T^. Model bacteria were cultured in LB broth for 24 h at 37 °C under constant agitation at 200 rpm. Fermentations were diluted to an optical density of 0.2 and then used to homogeneously inoculate LB agar plates. A drop of 10 µL of crude extract was placed over the Agar plates. Plates were then incubated for 18–24 h at 37 °C and inhibition zones were checked. A crude extract of ISP2-ASW medium and DMSO (10% v/v) were used as negative controls.

Crude extract dereplication was performed as previously described [77], employing a liquid chromatography-high resolution mass spectrometry (LC-HRMS) using Fundación MEDINA protocols [78]. For predominant components of the crude extract, molecular formulae, accurate masses, and UV spectra were obtained. To search for candidate molecules, the predominant components information was compared to Fundación MEDINA in-house database that harbour one the largest microbial collections and natural product libraries. Where no matches were obtained, a complementary search in the Dictionary of Natural Products of Chapman & Hall, a fully curated natural products database, was achieved.

## Results and discussion

### Genome assembly

**Fig. 1:**
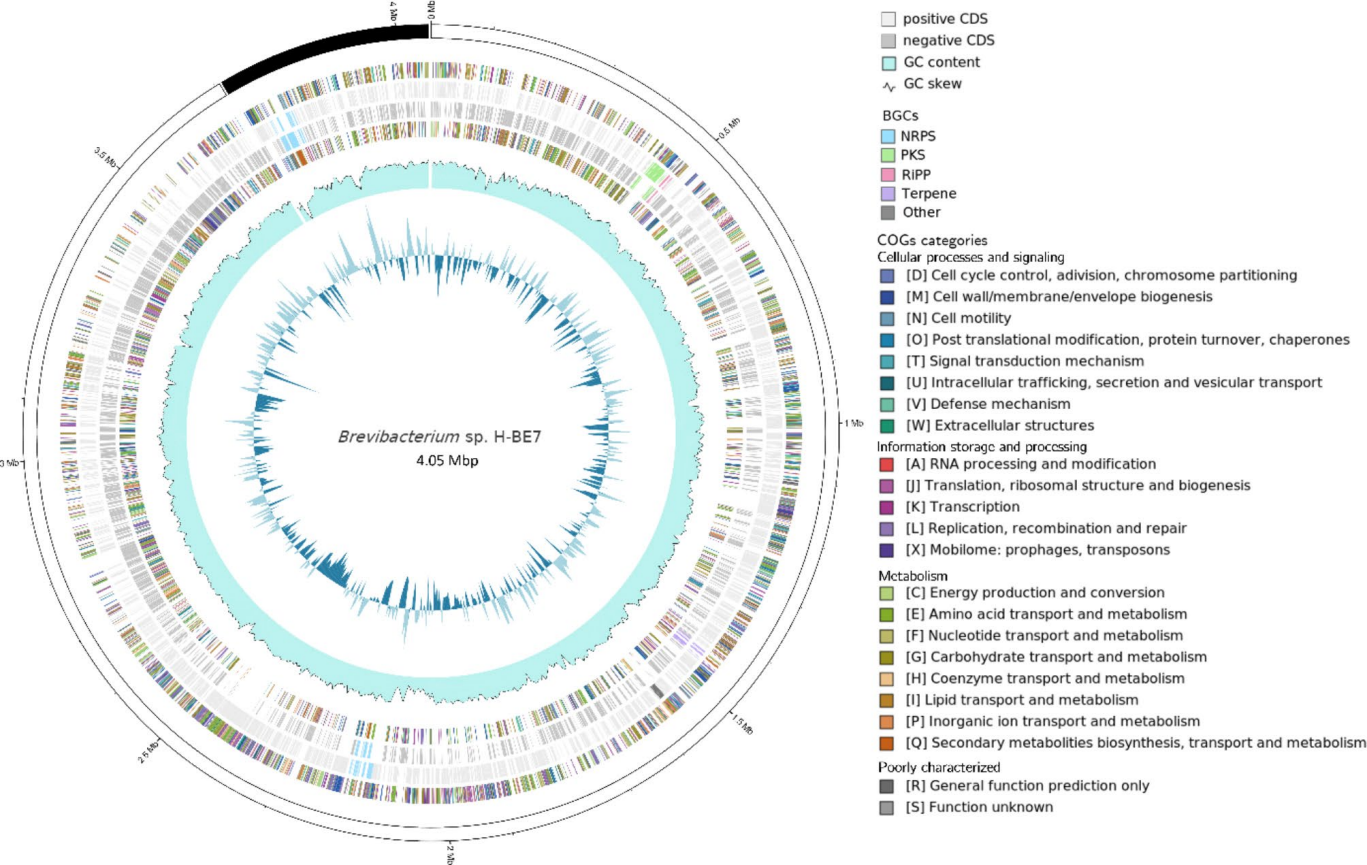
Circular genomic representation of *Brevibacterium* sp. H-BE7. From outside inward: contigs; CDSs coloured by COGs categories on the forward strand; CDSs with BGCs coloured on the forward strand: CDSs with BGCs coloured on the reverse strand; CDSs coloured by COGs categories on the reverse strand; GC content; GC skew

The genome of *Brevibacterium* sp. H-BE7 was sequenced using Illumina and ONT. Strain H-BE7 yielded a final assembly of 4.05 Mbp in two contigs, with a G + C content of 65.4% (Fig. 1). Genome size of *Brevibacterium* strains have been described to range from 2.3 to 4.5 Mbp, and have a G + C content from 58.0 to 70.9% [36], which makes the genome of strain H-BE7 a relatively large one for a species within the genus, but within average considering the G + C content.

According to the Cluster of Orthologous Genes (COGs) annotation, strain H-BE7 bears a large number of proteins classified as K (transcription), R (general function prediction only) and E (amino acid transport and metabolism), similar results were observed by previous reports [36, 79]. However, overall, there is scarce information on detailed COGs annotations for strains of species within *Brevibacterium* genus. Secondary metabolism biosynthesis, transport and metabolism, account for 2% of the predicted proteins of strain HBE-7.

AntiSMASH predicted six BGCs (Table 1), including two Non-ribosomal Peptide Synthetase (NRPS), one type three Polyketide Synthase (T3PKS), one RiPP, one Terpene and one Siderophore. Similarity to MIBiG repository BGCs [80] ranged from 0 to 75%, where only Terpene and Siderophore BGCs have more than 50% similarity (57% and 75% similarity to carotenoid and desferroxiamine E BGC, respectively). This data suggests that strain H-BE7 has the potential to produce novel natural products.

**Table 1:** Biosynthetic gene clusters (BGCs) of *Brevibacterium* sp. H-BE7.

*AntiSMASH type predictor*	Length (bp)	Predicted product	Similarity (%) ^a^	MIBiG-ID
T3PKS	41,362	5-acetyl-5,10-dihydrophenazine-1-carboxylic acid 5-(2-hydroxyacetyl)-5,10-dihydrophenazine-1-carboxylic acid endophenazine A1 endophenazine F endophenazine G	21	BGC0000934
RiPP-like	10,801			
Terpene	21,083	Carotenoid	57	BGC0000636
Siderophore	12,478	Desferrioxamine E	75	BGC0001478
NRPS-like	44,128	Ulleungmycin	5	BGC0001814
NRPS	50,620	Pepticinnamin E	10	BGC0002014

^a^ Percentage of genes from MIBiG BGCs showing nucleotide similarity to strain H-BE7 BGCs

The number of BGCs from *Brevibacterium* sp. H-BE7 is rather low when compared to other Actinomycetota larger genomes, such as those of *Streptomyces* or *Rhodococcus* [7]. Nevertheless, searching for non-*Streptomyces* strains, especially from environments with strong selective pressures, such as the ocean, has proven to be a successful strategy for bioprospecting [81–83].

### Phylogenomic analysis

To study the genomic potential of *Brevibacterium* for bioactive compounds synthesis, all 116 available *Brevibacterium* genomes (up to July 21st, 2021) were downloaded from NCBI. To prevent highly fractioned BGCs, only genomes with less than 200 contigs were downloaded. All genomes were analysed with CheckM, and eight genomes with completeness < 98% and contamination > 5% were discarded. Furthermore, to analyse the uniqueness of each genome, 10 redundant genomes were discarded, e.g., the same strain with different culture collection names. After all filters applied, 98 *Brevibacterium* genomes, including strain H-BE7 were selected for further analysis. To inspect if ecological niches reflect specific traits in *Brevibacterium* phylogeny, the isolation source for every genome analysed was retrieved. Overall, 98 strains originate from the following niches: aquatic (6); food (40); human (21); marine (11); other-unknown (12) and soil (8). As previously indicated, most strains of *Brevibacterium* species have been isolated from dairy products, especially from cheese [34–38]; however, several strains have been retrieved from human stool [28] or other human derived sources [27]. On the other hand, strain from environmental isolates represent 25.5% of *Brevibacterium* analysed, with several environments, such as aquatic [26], soil [23, 24] and marine ecosystems [29–33].

**Fig. 2:**
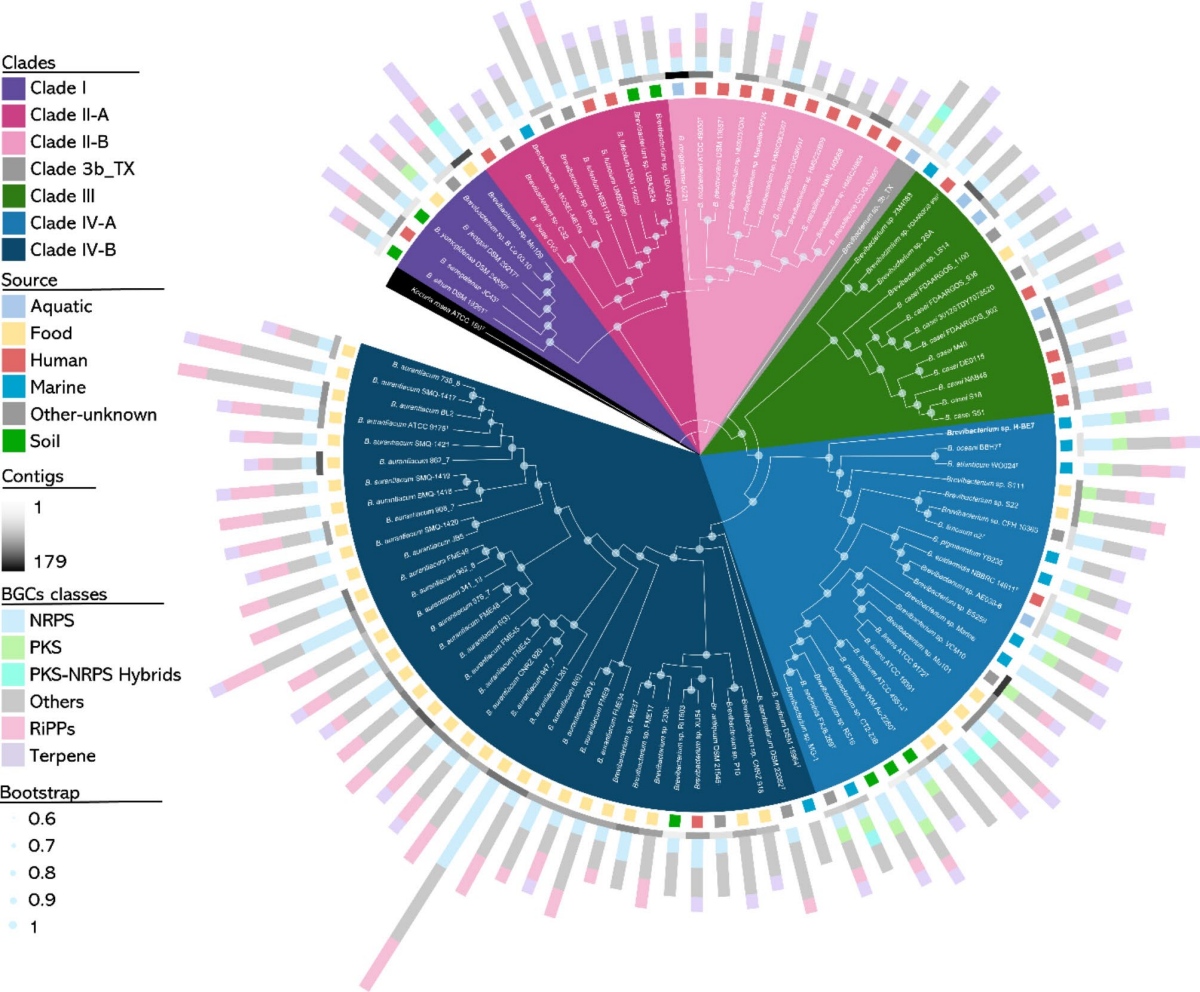
Phylogenomic cladogram of 98 *Brevibacterium* strains. Phylogeny is inferred, using Orthofinder v2.5.4, identifying 877 orthogroups present in all strains. Phylogenomic cladogram is visualized using iTOL. Clades are depicted in colours. Coloured squares beside strains represent isolation source and rectangles in greyscale indicate the number of contigs for each genome. Stacked bar chart indicates the number of BGCs for each strain coloured by BGC class. *Brevibacterium* sp. HBE-7, located within clade IV-B, is depicted in white bold font. Bootstraps are indicated in each branch as a light-blue circle. *Kocuria rosea* ATCC 186^T^ was used as outgroup

A phylogenomic cladogram was inferred, using Orthologue analysis approach. *Brevibacterium* strains are divided into four major clades (Table 2), except for *Brevibacterium* sp. 3b_TX, which forms a distinct branch between clades IIB and III (Fig. 2). Clade I has the least number of representatives, with six strains from four niches. Clade II is subdivided into clade II-A, where *B. ihuae* and *B. luteolum* strains are present, and clade II-B, where most of the strains have been isolated from human sources (90.9%). Clade III is represented predominantly by strains of *B. casei* (69.2%), isolated from various sources, but mostly from human-derived niches. Clade IV is the largest clade and is subdivided into clade IV-A, with eight of the 11 marine-derived *Brevibacterium*, including strain H-BE7. Within clade IV-A, *B. iodinum* ATCC 49514^T^ [84] is included, which is a producer of phenazines [85], and *B. linens* strains, of the same species as *B. linens* M18, producer of Linocin M18, with no genome available [39]. Clade IV-B is mostly characterized by *B. aurantiacum* strains, which account for up to 26.5% of all *Brevibacterium* from this study. Most strains from this clade (86%), including all *B. aurantiacum* strains, have been isolated from dairy products. With the exceptions of clade II and strain 3b_TX, food related *Brevibacterium* are present in each clade of the phylogenomic cladogram. Most marine *Brevibacterium* are present in clade IV (Table 2).

**Table 2:** Summary of *Brevibacterium* phylogenomic clades

Clade	#Strains	#Marine strains	Most common Isolation source	Average #BGC per strain
I	6	0	Soil and Food	5.6
II-A	9	1	Other-unknown	4.4
II-B	11	0	Human	3.2
3b_TX	1	0	aquatic	5
III	13	1	Human	4.6
IV-A	22	8	Marine	6.1
IV-B	36	1	Food	6.6
Total	**98**	**11**	**Food**	**5.5**

BGCs were predicted from all strains, using antiSMASH. Overall, all 98 strains have 543 BGCs classified in the following categories: NRPS, PKS, PKS-NRPS hybrids, RiPPs, terpenes and Other BGCs. No strong correlation between the number of contigs and the number of BGCs was observed when applying a linear regression (Fig S1.A). The abundant categories included, Other BGCs (43.8%), followed by NRPS (20.3%), RiPPs (17.5%), terpenes (14.4%), PKS (3.1%) and PKS-NRPS hybrids (0.9%). An average of 5.5 BGCs per strain were predicted, with *B. aurantiacum* L261 exhibiting the largest number of BGCs (17); whereas three closely related strains *B. massilience* CCUG 56047, *Brevibacterium* sp. HMSC22B09 and *B. massilience* NML 140868 located in clade II-B, contained the fewest number of BGCs (2 each). BGCs distribution patterns could be observed within phylogenomic clades (Fig. 2), becoming evident that the number of BGCs differ, being clade IV-B, the clade with the highest number of BGCs per strain (6.6), followed by clade IV-A (6.1). The clade with fewer BGCs per strain is clade II-B (3.2) (Table 2). The prevalence of genome reduction in mutualistic and symbiotic bacteria can potentially explain the observed reduction in genome size within most strains from clade II-B, which are primarily human-derived, contrasting with strains from clade IV [86]. The proportion of NRPS contrasted with the total number of BGCs is slightly higher within clade II-A (25%) (Figure S1.B), and lowest in clade II-B (14.3%), whereas the number of RiPPs are higher in clade IV-B (25.8%), contrasting with clade II-A and clade III (5%). Terpenes range from 20% in clade III to 32.4% in clade I, although, in clade IV-A and IV-B they are less frequent, with 13.5% and 7.2% respectively. Finally, PKS are present only in clade IV-A (2.3%) and in one strain from clade I (*B. jeotgali* DSM 29217^T^) and *Brevibacterium* sp. 3b_TX.

### Average nucleotide identity analysis

**Fig. 3:**
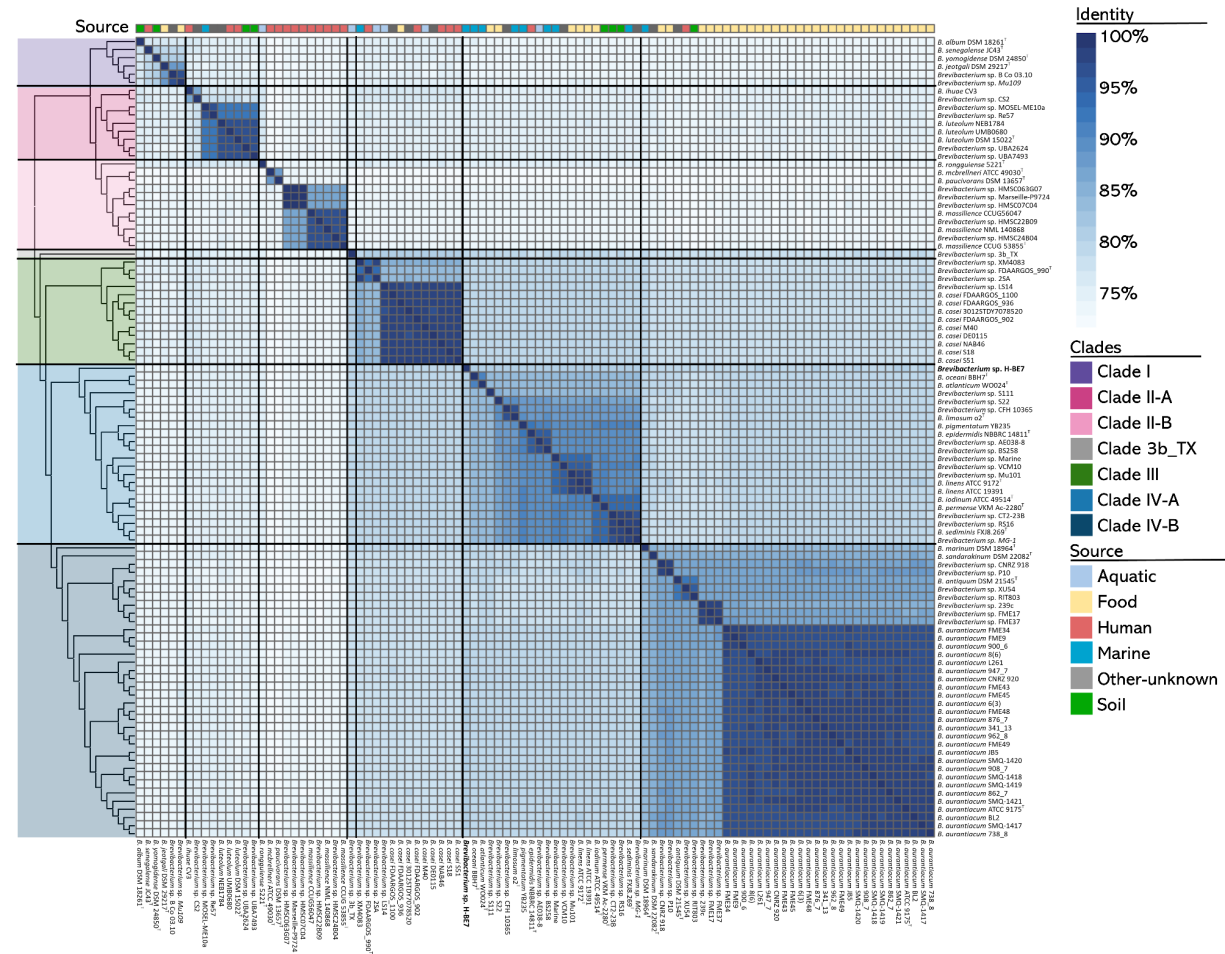
BLAST based Average Nucleotide Identity (ANIb) of 98 *Brevibacterium* strains. ANIb was calculated, using PyANI. Clades are depicted in colours. Coloured squares beside strains represent isolation source

An ANIb analysis was calculated for comparing every strain’s similarity within each other (Fig. 3). PyANI was employed to perform BLAST searches using 1 kb genomic fragments as queries against a target genome [72]. In this study, ANIb values were calculated for all pairwise comparisons between strains. Results indicate the presence of several potentially new species of *Brevibacterium*, when applying an ANIb value of more than 95% as the intra-species range [72, 87]. Nevertheless, it is worth noting that there are exceptions to this threshold, and higher taxonomic ANIb thresholds remain to be precisely defined [72, 87]. *Brevibacterium* sp. B Co 03.10, uploaded to NCBI as *B. yomogidense*, shares an ANIb of 80.6% with *B. yomogidense* DSM 24850^T^, suggesting that it does not belong to that species. Strains B Co 03.10 and Mu109 group together within clade I, with an ANIb of 96.5%, and could represent the same species. In clade II-A, all three *B. luteolum* strains exhibited an ANIb of 95.5% or higher with each other and with *Brevibacterium* sp. UBA2624 and *Brevibacterium* sp. UBA7493, suggesting that both strains could belong to that species. *Brevibacterium* sp. MOSEL-ME10a has been previously characterized as *B. luteolum* [88], however with *B. luteolum* strains, it shares an ANIb as high as 92.6%, therefore, it is improbable that strain MOSEL-ME10a belongs to the species *B. luteolum*. From clade II-B, *Brevibacterium* sp. HMSC063G07, *Brevibacterium* sp. Marseille-P9724, and *Brevibacterium* sp. HMSC07C04 could be strains from the same new species, having an ANIb of 98.6% or higher with each other. Additionally, *B*. *massilience* strains have an ANIb of, at least, 95.9% with *Brevibacterium* sp. HMSC22B09 and *Brevibacterium* sp. HMSC24B04, which could indicate that both strains belong to that species.

*Brevibacterium* sp. 3b_TX is the most divergent strain among all 98 *Brevibacterium*, grouping in a distinct branch of the cladogram; the maximum ANIb is shared with *B*. casei FDAARGOS_1100 (80.4%). Strain 3b_TX was uploaded to NCBI as *B. celere*, however, the taxonomy check is inconclusive, and no other *B. celere* genomes were available to compare. Observing a 16S rRNA phylogenetic tree inferred by Levesque et al., 2019, strain 3b_TX does not group with other *B*. *celere* strains, such as *B. celere* KMM 3637^T^ [89], suggesting that it could be a new species within *Brevibacterium*. In clade III all *B. casei* have an ANIb of at least 97.1% between each other. Altogether, *Brevibacterium* sp. LS14 shares a high ANIb similarity with strains of the *B. casei* species, suggesting that it could also belong to that species. Observing clade IV-A, several strains could potentially be new *Brevibacterium* species, including strain H-BE7, which major values of ANIb are 81.2% and 81.3% with *B*. *oceani* BBH7 ^T^ [30] and *B*. *atlanticum* WO024^T^ [29] respectively, both isolated from marine sediments. Altogether, *B. limosum* o2^T^, isolated from marine sediments, shares an ANIb of 96.3% with *Brevibacterium* sp. CFH 10365 isolated from the grass carp intestine, suggesting that the latter could belong to *B. limosum* species. *Brevibacterium* sp. Mu101, *B. linens* ATCC 9172 ^T^, and *B. linens* ATCC 19391 share an ANIb of 97.6% or higher, indicating that strain Mu101 could be part of *B. linens* species. The marine strain *B. sediminis* FXJ8.269 ^T^ shared an ANIb of at least 97.1% with *Brevibacterium* sp. CT2-23B, *Brevibacterium* sp. RS16, and *Brevibacterium* sp. MG-1, therefore these strains could be part of that species. Clade IV-B is quite homogenous with several *B. aurantiacum* strains which share an ANIb of at least 96.2%. Within this clade, some strains could be new species in the *Brevibacterium* genus, such as strain CNRZ918, together with P10, strain 239c together with FME17, and FM37, and finally, strain RIT803 and XU54 [90], each could be new species.

Remarkably, 25 strains have ANIb values of less than 95% with any other *Brevibacterium*, indicating they could be unique new species within *Brevibacterium* genus. Of those strains, two were isolated from aquatic niches, three from other-unknown, four from soil, four from food, six from human, and six from marine samples, which indicates the importance of searching for *Brevibacterium* in environmental ecosystems to retrieve all its diversity. These results show that there are several new species of *Brevibacterium* in other ecosystems, since 12 of 25 environmentally isolated strains (aquatic, marine, or soil), could potentially be unique new species. It is important to note, that 38 validly published entries for *Brevibacterium* species according to the List of Prokaryotic names with standing in Nomenclature (LPSN) [91]. However, it should be noted that within this study 23 species are included, therefore some of the strains identified as potentially new species may have already been described. This could be due to their genomes not being available or not being included in the study.

### Pangenome analysis

A pangenomic analysis of 98 *Brevibacterium* strains was performed, using anvi’o pangenomic workflow. Anvi’o identifies amino acid sequences and groups them by similarities into gene clusters. A total of 26,630 gene clusters were found in all strains, where 656 are present in the core genome or the gene clusters present in all strains; 564 gene clusters represent the soft core, present in 81 to 87 genomes; 2,076 the shell genome, from 28 to 80 genomes; 1,760 the soft shell, from 11 to 27 genomes; 11,144 the cloud genome, from two to 10 genomes; and 10,430 the singletons, or unique gene clusters (Figure S2). Approximately 2.5% of gene clusters represent the core genome of *Brevibacterium*, and, altogether, less than 5% of gene clusters are present in 81 or more genomes. On the other hand, 39% of gene clusters are found in singletons that, in addition to cloud genes, represent 80% of total gene clusters. Similar results were obtained when analysing 23 *Brevibacterium* genomes, where only 1% of gene clusters represent the core genome of this group [36]. Another study analysed the pangenome of 11 *B. aurantiacum* strains, to find that 41.7% of their gene clusters are either conserved in all genomes with 28.6% specific to one strain [34]. Pangenome analysis of genetically close strains show a higher number of gene clusters present in the core genome, especially at the species level [92]; therefore the selection of strains for pangenome analysis is fundamental. Few studies consider the completeness of genomes for pangenome analysis. Consequently there is an underestimation of sequence variation in bacteria, caused by short regions that accumulate rapid changes and are hard to assemble from short-reads [93].

Functional analyses were conducted to assess the enrichment or occurrence of specific gene clusters within a particular group of strains based on their phylogenomic clades or isolation source. The evaluation focused on identifying enriched functional traits associated with COGs. However, niche-enriched functions were disregarded in this analysis, as the correlation between phylogenomic clades and COG functions was deemed more meaningful and informative. Some specific functions for clade I, include a ribosome-binding protein aMBF1, a transcriptional coactivator [94]. Clade II is enriched in the 4-alpha-glucanotransferase MalQ, which is essential for the metabolism of maltose and the degradation of maltodextrins [95]. Clade III strains are exclusively enriched with H+/Cl- antiporter ClcA [96] and Co/Zn/Cd cation transporters. With exception of *Brevibacterium iodinum* ATCC 49514 ^T^, clade IV is enriched in transcriptional regulator LsrR [96]. Additionally, clade IVA is enriched in 3-hydroxy-3-methylglutaryl CoA synthase, involved in the mevalonate pathway and isopentenyl pyrophosphate production. The principal end product of isopentenyl pyrophosphate metabolism includes the lipid carrier undecaprenol, menaquinones, phenazines, and carotenoids [97].

### BGC diversity and distribution among Brevibacterium phylogenomic clades

.

**Fig. 4:**
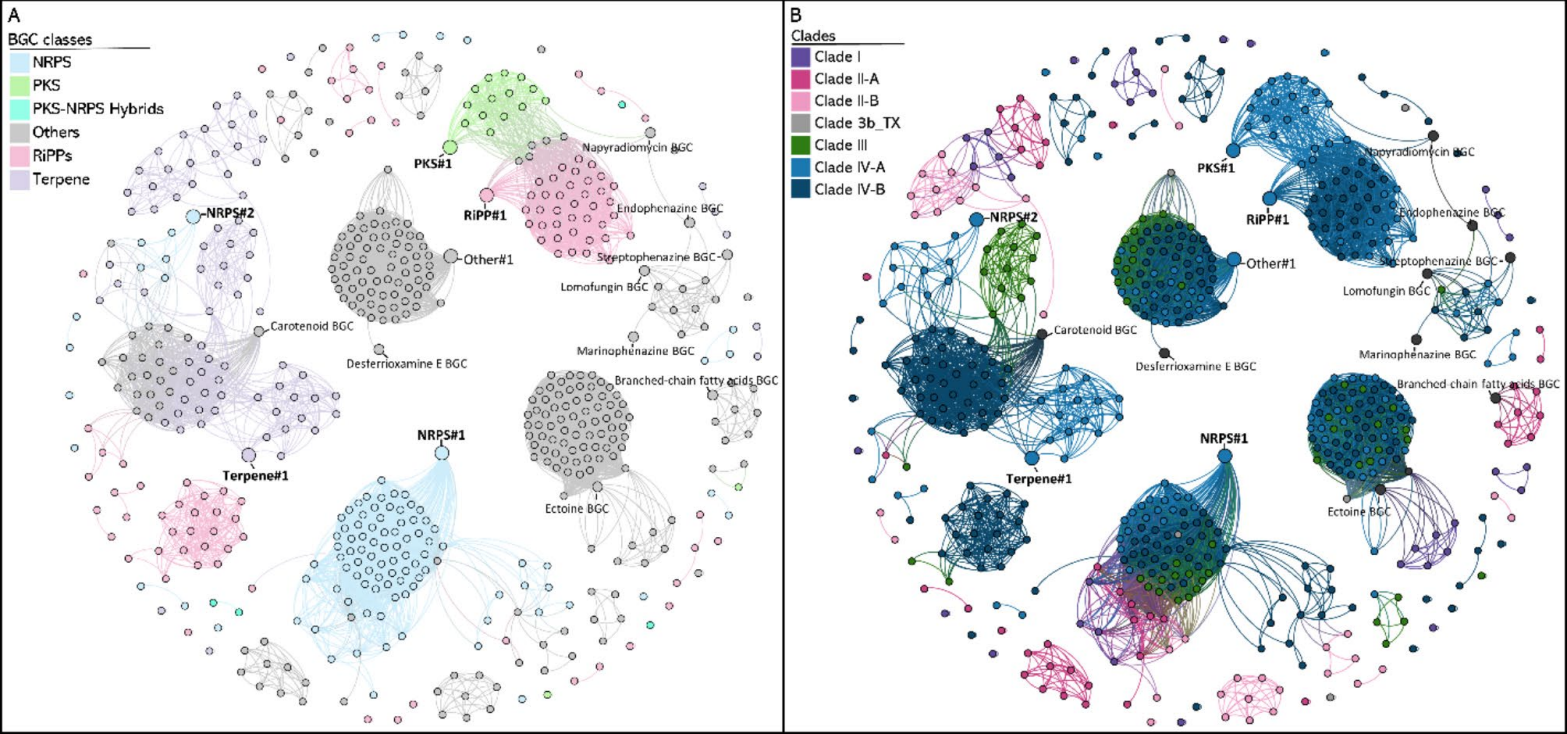
**Brevibacterium BGC networking**. The distance network was constructed based on the BGCs of 98 *Brevibacterium* genomes, leading to a total of 543 BGCs. Each node represents a single BGC, connected when sharing a BiG- SCAPE raw distance cut-off of ≤ 0.6. *Brevibacterium* sp. H-BE7 BGCs are displayed, numbered according to their BGC class, and shown apart from the main group of nodes, but maintaining their connections. MIBiG BGCs are also displayed in the network and labelled according to their product. (**A**) Nodes are coloured by BGC classes. (**B**) Nodes are coloured according to phylogenomic clades

Genome mining of 98 *Brevibacterium* genomes predicted 543 BGCs with antiSMASH v6.0.1. Most strains of *Brevibacterium* species from every phylogenomic clade harbour NRPS, Terpene and RiPP BGCs (Fig. 2). To obtain an overview of the BGC diversity among *Brevibacterium*, a BiG-SCAPE sequence similarity BGC network was constructed (Fig. 4). Some groups of BGCs connect with MIBiG repository BGCs, such as carotenoid, desferrioxamine, ectoine, and phenazine-related BGCs. However, several BGCs group without connecting with MIBiG BGCs, exhibiting *Brevibacterium* BGCs with unknown products. Some groups of *Brevibacterium* BGCs present a balloon-shaped structure, connecting only within a specific category, however, most BGCs connect with other classes.

The same network was coloured according to phylogenomic clades to analyse the distribution of groups of BGCs among phylogeny (Fig. 4.B). It is possible to observe some specificity among the groups of BGCs, such as the siderophore group of BGCs connecting with desferrioxamine E from MIBiG (BGC0001572), which is present within clade III, clade IV, and strain 3b_TX. Desferrioxamines are widely distributed among soil and aquatic bacteria [98], and plays a central role in iron metabolism of cheese microbial communities. This is consistent with low iron content in these environments, enabling these microorganisms dwelling within them to produce siderophores to improve iron acquisition [36].

Altogether, a group of other class BGCs, Terpenes and NRPS, groups with a carotenoid BGC from MIBiG (BGC0000636), and it is included in clade III and clade IV, with the exception of one BGC from strains in clade II-A. Carotenoids have been isolated previously from *Brevibacterium* strains [34, 90]. This is consistent with the high presence of cheese-related strains within those clades, where carotenoids confer key organoleptic properties. Moreover, there is a group of BGCs exclusively from clade II-A which groups with a branched-chain fatty acid BGC (BGC0001534) from MIBiG, which harbour genes that encode branched-chain amino acid dehydrogenase and a transcriptional regulator previously reported to play a role in the synthesis in daptomycin analogues in *Streptomyces roseosporus* [99].

Some BGCs are conserved among *Brevibacterium*, such as a NRPS BGC, with 94 BGCs belonging to 80 strains within all phylogenomic clades, although this family does not connect with any MIBiG BGC; therefore, their product is still unknown. Another conserved BGC is an ectoine BGC, which is present within the genome of 85 of 98 strains. Previous studies already reported the presence of ectoine BGCs in *Brevibacterium* [36]. Ectoine are protective compounds that help bacteria survive under osmotic stress [100], for example helping them to survive in saline environments such as cheese and marine ecosystems. This osmolyte is synthesized from the precursor L-aspartate-β-semialdehyde, a key intermediate in the metabolism of microbial amino acids and cell wall synthesis. The biosynthetic pathway involves a sequential action of enzymes: L-2,4-diaminobutyrate transaminase (EctB), L-2,4-diaminobutyrate acetyltransferase (EctA), and ectoine synthase (EctC) [101, 102]. Clustering among ectoine BGCs in *Brevibacterium* vary depending on the phylogenomic clade. Ectoine BGCs from clades I, III, IV, 3b_TX are connected with an ectoine BGC from MIBiG repository (BGC0000852); which contains the clustered *ectABC* genes. However, ectoine BGCs from clade II-B are connected to a different group due to the loss of *ectA* and *ectB* genes. Strains of this clade (isolated from human samples), and those lacking the complete BGC (isolated from various niches, such as soil, cheese, and marine niches), may not require the biosynthesis of this osmolyte and might rely on different survival mechanisms.

Interestingly, a clade-specific BGC can be observed. According to antiSMASH results, most strains in clade IV-A possess a unique PKS BGC not present in the rest of the genomes. Except for strains *Brevibacterium* sp. 3b_TX and *B. jeotgali* DSM29217 ^T^, which also harbour a PKS BGC (clade I), clade IV-A possesses a unique group of PKS BGCs. Similar features were observed in species of the *Pseudoalteromonas* genus, where PKS-NRPS hybrids are exclusively in the genome of a single clade within the genus, which additionally harbours a larger number of BGCs [103]. In addition, another study observed the presence of a species-specific PKS BGC within *Rothia kristinae*, with some similarity to endophenazine BGC [104].

From the total, 28 BGCs represent singletons in the network (5.2%), indicating that only a small proportion of *Brevibacterium* BGCs represent relatively recent acquisition events [105]. When observing the same BGC network coloured by isolation source, there is no clear correlation between niche and BGCs (data not shown).

The clade-specific PKS BGC group is connected to a group of RiPPs through five BGCs that are classified as Other by antiSMASH (Fig. 4.A). This connection extends to napyradiomycin BGC (BGC0001079) from MIBiG which in turn connects with BGCs encoding phenazines: endophenazine (BGC0000934), streptophenazine (BGC0002010), marinophenazine (BGC0001221), and lomofungin (BGC0001302) BGCs. Remarkably, PKS class BGCs are present only in strains belonging to clade IV-A, excepting strain *B. jeotgali* DSM 29217 ^T^ and *Brevibacterium* sp. 3b_TX (Fig. 2), which have a PKS BGC that does not share similarities with PKS BGCs in strains from clade IV-A. The distribution pattern of BGCs in *Brevibacterium* follows a phylogenetic pattern similar to that observed in the *Rhodococcus* genus, where BGC families are determined by phylogeny rather than niche [7].

**Fig. 5:**
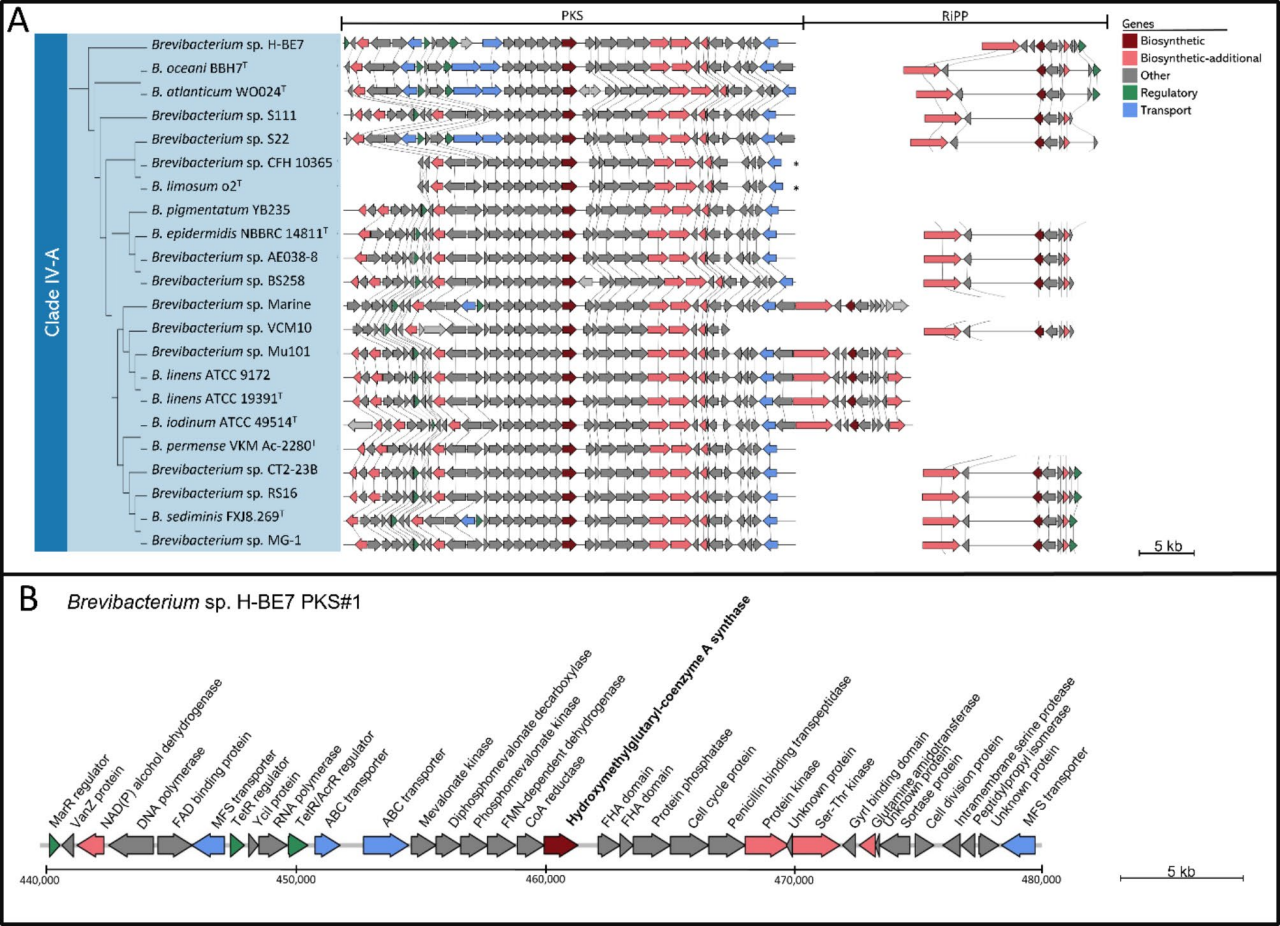
Distribution of PKS BGC in *Brevibacterium* strains. (**A**) BGCs are displayed next to the phylogenomic tree from clade IV-A. (**B**) Genetic representation of PKS#1 BGC from *Brevibacterium* sp. H-BE7. Each gene predicted function by BLASTP is displayed above the gene. In each section, genes are drawn according to the size bar. * represent BGCs not predicted by antiSMASH.

Upon examining the PKS BGCs from clade IV-A, we noted a connection with another group of BGCs of RiPP class. Both groups were linked by five BGCs categorized as Other. Therefore, a deeper insight into these BGCs was accomplished. Using antiSMASH, we predicted the presence of a PKS BGC in all strain of clade IV-A, except for seven strains. To confirm this uniqueness to clade IV-A and its absence in other strains of the phylogenomic tree, we utilized the CORASON tool [74], and successfully detected this PKS BGC in all strains within clade IV-A (Figure S3). Additionally, antiSMASH results predicted the presence of RiPP BGCs in almost every strain from clade IV-A, as well as in every *B. aurantiacum* strain from clade IV-B. To identify additional BGCs that were not predicted by antiSMASH, we employed the Cblaster tool [106] across all *Brevibacterium* strains. This search yielded a total of seven additional PKS and three RiPP BGCs. To analyse the similarities between these BGCs, we utilized the Clinker tool [76] (Fig. 5, Figure S4).

The PKS BGC in clade IV-A appears to be highly conserved (Fig. 5.A). Interestingly, two PKS BGCs from strain CFH 10,365 and o2^T^ were not predicted by antiSMASH and were predicted using CORASON and Cblaster. Additionally, five strains within this clade, possess a hybrid BGC with biosynthetic genes belonging to two distinct types of BGCs, a PKS and a RiPP. These hybrid BGCs are classified as Other by antiSMASH. The RiPP section of those BGCs is similar and highly conserved between strains from clade IV-A, as well as some strains from clade IV-B (Figure S4). Therefore, those five BGCs were responsible of the link between the PKS and RiPP family of BGCs in the network (Fig. 4.A). Upon examining the *Brevibacterium* sp. H-BE7 genome (Fig. 1), it is apparent that the PKS and RiPP BGCs are adjacent in the genome. This is also observed in the genomes of other strains (data not shown), which suggests that the difference between the five BGCs classified as “Other” in clade IV-A is due to antiSMASH predictions. These results emphasize the importance of using different tools when analysing the presence of BGCs within groups of bacteria.

Furthermore, all *B. aurantiacum* strains in clade IV-B possess the same RiPP BGC observed in clade IV-A. Only three other strains from clade IV-B have a similar BGC within their genomes. However, these three BGCs lost the biosynthetic core gene of the cluster, therefore were not predicted by antiSMASH (Figure S4). The biosynthetic gene from the RiPP#1 BGC of *Brevibacterium* sp. H-BE7 has a sequence identity of 98.9% with the linocin M18 gene from *B. linens* M18 [107]. However, the complete BGC does not match with any known BGC from MIBiG.

### Insights into the clade specific PKS and RiPP BGCs

When analysing the BGC network (Fig. 4), the group of PKS and RiPP BGCs shows connections with the napyradiomycin BGC (BGC0000652) [108]. These connections are based on four genes from the PKS BGC, including the biosynthetic PKS gene and three genes categorized as other by antiSMASH. By examining KnownClusterBlast analysis [109] of antiSMASH results for the PKS#1 BGC of strain H-BE7, it is determined that the biosynthetic PKS gene encodes a hydroxymethylglutaryl-coenzyme A synthase (Fig. 5.B). This gene shares 48% of aminoacid similarity with the *hmgr* gene from endophenazine A and B in *Streptomyces anulatus* 9663 (BGC0001080) [110, 111] from MIBiG.

Furthermore, four downstream genes, that encode a CoA reductase, FMN-dependent dehydrogenase, phosphomevalonate kinase, and diphosphomevalonate decarboxylase, exhibit similarities to the phenazine A BGC, corresponding to the *ippi*, *pmk*, *mdpd* and *mk* genes, respectively. The five genes that have similarities to phenazine BGCs, are involved in the mevalonate pathway [45, 111], which agrees with the functional analysis of the pangenome, where clade IV-A is enriched in 3-hydroxy-3-methylglutaryl CoA synthase involved in the mevalonate pathway.

To examine the genetic distribution of this BGC among *Brevibacterium*, the CORASON tool [74] was utilized with the CoA reductase gene from PKS#1 as query (Figure S3). The resulting phylogenetic tree created by CORASON exhibits a similar distribution of the BGC within the clade, resembling the pattern observed in the phylogenomic cladogram (Fig. 2). This similarity suggests a shared ancestral origin for the BGC, implying that vertical gene transfer may have played a significant role in the evolution of this phylogenomic-dependent BGC [7, 105].

Notably, in a recent comparative genomic study of the *Rothia* genus, it was discovered that *Rothia kristinae* possesses a species-specific PKS BGC that shows similarities to endophenazine BGC found in the MIBiG database [104]. Interestingly, this species of *Rothia* is the only one identified to have a PKS BGC, which parallels the pattern observed in our study. To investigate further, we looked into the genomes of *R*. *kristinae* and compared their PKS BGCs to that of strain H-BE7 using the Clinker tool (Figure S5). Our analysis reveals that these BGCs share similarities in four genes encoding: hydroxymethylglutaryl-coenzyme A synthase; CoA reductase; FMN-dependent dehydrogenase; and phosphomevalonate kinase. However, no additional phenazine core biosynthetic genes were found. It is noteworthy that a similar phenomenon observed in the *Brevibacterium* genus is also observed within the *Rothia* genus, where only one specific clade possesses a PKS BGC with genes associated with the mevalonate pathway and shared with phenazine BGCs. The findings indicate that the presence of these genes in specific clades of both the *Brevibacterium* and *Rothia* genera may be attributed to a process involving horizontal gene transfer, followed by the vertical transmission and preservation of these genes within the descendant lineages [105].

Phenazine-related biosynthetic genes were found in nine distinct *Brevibacterium* strains, including *B. casei* 3012STDY7078520, *Brevibacterium* sp. S22, *B. linens* ATCC 9172^T^, *B. iodinum* ATCC 49514^T^, *Brevibacterium* sp. P10, and *B. aurantiacum* L261, ATCC 9175^T^, BL2 and SMQ-1417. These strains were identified to have a predicted phenazine biosynthetic gene cluster (BGC), based on antiSMASH analysis, along with the presence of *ppzB, ppzD, ppzE, ppzF and ppzG* from *S. anulatus* 9663, involved in phenazine core biosynthesis [48]. Remarkably, these nine strains show no phylogenetic relatedness and belong to clades III, IV-A, and IV-B. The presence of these phenazine biosynthetic genes was investigated in all *Brevibacterium* strains, using CORASON and local BLAST searches, confirming that only these nine strains possess these phenazine genes in their genomes. Specifically, strains from clade IV-A, such as *Brevibacterium* sp. S22, *B. linens* ATCC 9172^T^ and *B. linens* ATCC 49,514, harbour both the phenazine BGC and the PKS associated with the mevalonate pathway. All nine strains have been isolated from food/related ecosystems, primarily cheese, except for strain 3012STDY7078520 of unknown origin.

### Antimicrobial activity and chemical dereplication

*Brevibacterium* sp. H-BE7 EtOAc crude extracts from 10-day cultures exhibited inhibition zones in *S. enterica* [51]. In this study, we proved the same inhibition, as well as inhibition against the food pathogen *L. monocytogenes* was observed, using the same growth conditions.

To analyse the chemical nature of the compounds present, a chemical dereplication was achieved, using LC-HRMS. The masses and the UV spectra of the most predominant peaks present in the crude extract were compared with the internal database of Fundación MEDINA. In the crude extract, coincidences with diketopiperazines were observed, molecules that are frequently found in bacterial cultures and could be acting as signalling molecules [77, 112]. Other matches include 2-quinolinylmethanol, with non-biological activity previously reported; lumichrome, a known degradation product of riboflavin with plant growth promoter activity, and 1-methoxyphenazine, which has antibacterial activity reported, in high doses (1 mg ml^− 1^), against *S. aureus, E. coli, P. aeruginosa* and *Salmonella typhi* [113]. Phenazines have been previously reported to be produced by *Brevibacterium*, such as the marine *Brevibacterium* sp. KMD 003 [41], and the milk-derived *B. iodinum* ATCC 49514^T^ [36, 42], and could be responsible for the antibacterial activity observed in strain H-BE7.

Dereplication of H-BE7 crude extract suggests that the antibacterial activity could be derived from a phenazine-like compound. However, since strain H-BE7’s genome presents non-characterized BGCs, and a phenazine-like BGC is not present, it is possible that the activity could be due to a compound produced by any of the BGCs present. *Brevibacterium* strains have been widely isolated from dairy products, conferring key organoleptic features and pigments [34, 36]. Additionally, antibacterial compounds have been identified in cheese-related *Brevibacterium*, such as the bacteriocin Linocin M18, from *B. linens* M18, which inhibits the growth of *Listeria* spp. [39]. Putative bacteriocin gene clusters have been found in cheese-associated *Brevibacterium*¸ which might give ecological advantages to bacteria related to this ecosystem [36]. The marine *Brevibacterium* sp. H-BE7 showed antibacterial activity against known food pathogens, revealing that antimicrobial compounds could bring ecological advantages to *Brevibacterium* inhabiting other ecosystems. Most *Brevibacterium* studies involve the analysis mostly of cheese-related strains, and little on strains from other environments [34, 36], hindering ecological-related inferences. Searching for *Brevibacterium* in other ecosystems, would help make stronger ecological analysis, and could render in the discovery of new antibiotic-producing strains, which could give other antibacterial advantages and possibly new organoleptic features to the food industry.

After finding the presence of 1-methoxyphenazine within the crude extract of *Brevibacterium* sp. HBE7, core phenazine biosynthetic genes were searched among the 98 *Brevibacterium* strains, using antiSMASH, CORASON and BLAST. Notably, although 1-methoxyphenazine is detected in the crude extract of strain H-BE7, its genome lacks the *ppzB, ppzD, ppzE, ppzF and ppzG* genes, indicating the presence of an unknown biosynthetic pathway for this compound. It is uncommon for researchers to investigate homologous systems once a biosynthetic gene cluster directing the synthesis of an active metabolite has been discovered [50], hence the synthesis of different biosynthetic pathways for phenazines remains unexplored. However, this phenomenon could be explained by the presence of genes with similar functions to *ppzB, ppzD, ppzE, ppzF and ppzG* within H-BE7’s genome, or from shared biosynthetic machinery with promiscuous enzymes [50, 114].

## Conclusion

*Brevibacterium* exhibits diverse biosynthetic capabilities and show great potential for bioprospecting, particularly in the search for novel antimicrobial compounds. The distribution of biosynthetic gene clusters varies among phylogenomic clades and some clade-specific BGCs connect with known BGCs in related pathways. Notably, strains of *Brevibacterium* genus possess the ability to synthesize bioactive compounds, which could provide them with a competitive advantage in their respective environments, such as cheese. Furthermore, the presence of uncharacterized BGCs in *Brevibacterium*, along with the likelihood of discovering new species in non-dairy environments, makes them a promising source for novel antibacterial compounds. The identification of a phenazine-like compound in the crude extract of strain H-BE7, despite the absence of core phenazine biosynthetic genes, suggests the existence of alternative biosynthetic pathways or promiscuous enzymes within its genome. Exploring *Brevibacterium* strains from diverse environments holds the potential to unveil new species and biosynthetic pathways, contributing to our understanding of their ecological and biotechnological significance.

### Electronic supplementary material

Below is the link to the electronic supplementary material.

Supplementary Material 1

Supplementary Material 2

Supplementary Material 3

Supplementary Material 4

Supplementary Material 5

Supplementary Material 6

## Data Availability

The genome of *Brevibacterium* sp. H-BE7 is deposited in GenBank/ENA/DDBJ under GCA_030227105.1 accession number and the reads at the SRA under the accession number PRJNA977705.
