# Supplementary figures and images for: Exploring the biosynthetic gene clusters in Brevibacterium: a comparative genomic analysis of diversity and distribution
Source: BMC Genomics. 2023 Oct 19;24:622. doi: 10.1186/s12864-023-09694-7 (PMC10588199; doi:10.1186/s12864-023-09694-7)

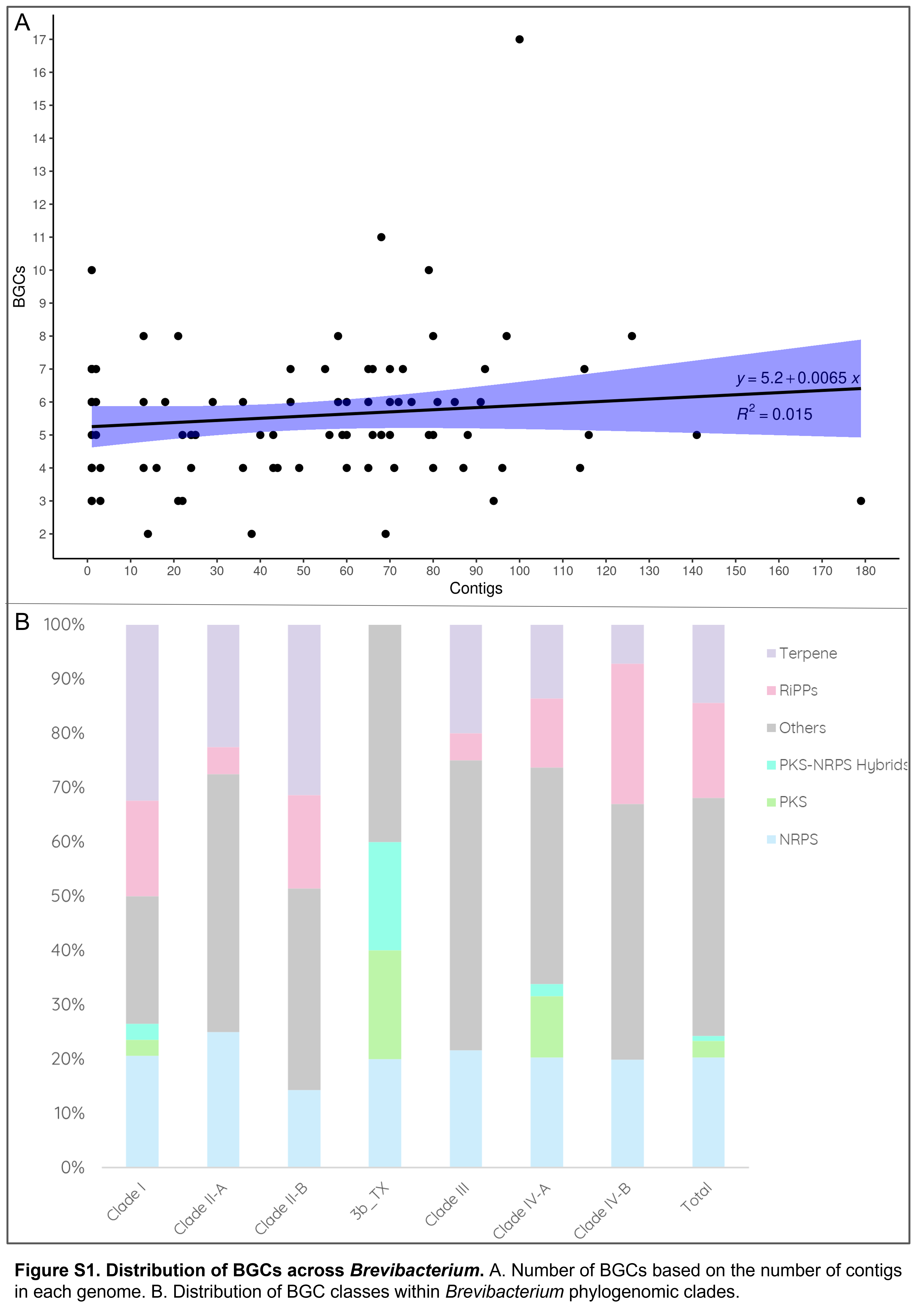

Supplement: Supplementary file 1 — Supplementary Material 1 [file 12864_2023_9694_MOESM1_ESM.png]

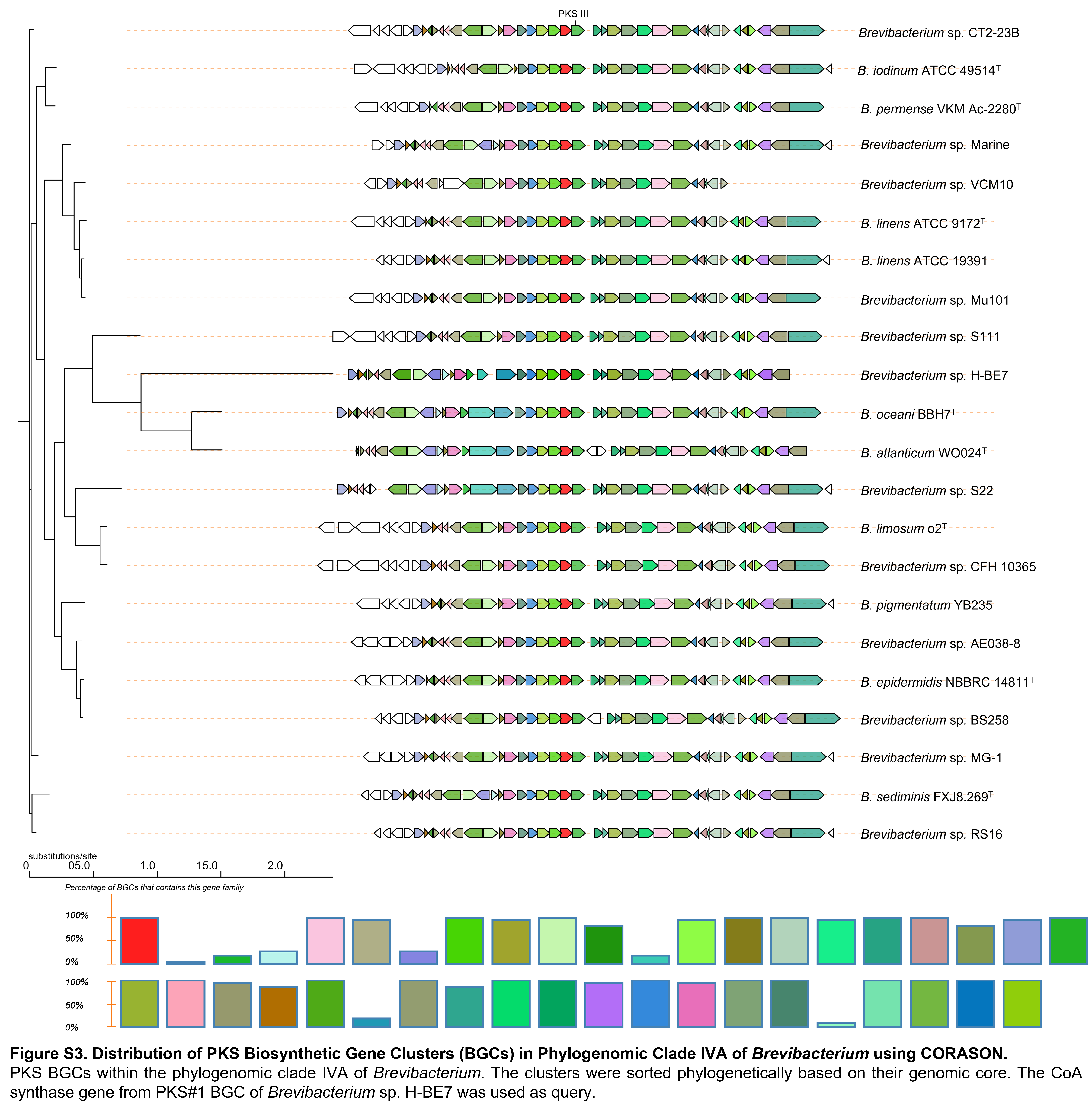

Supplement: Supplementary file 2 — Supplementary Material 2 [file 12864_2023_9694_MOESM2_ESM.png]

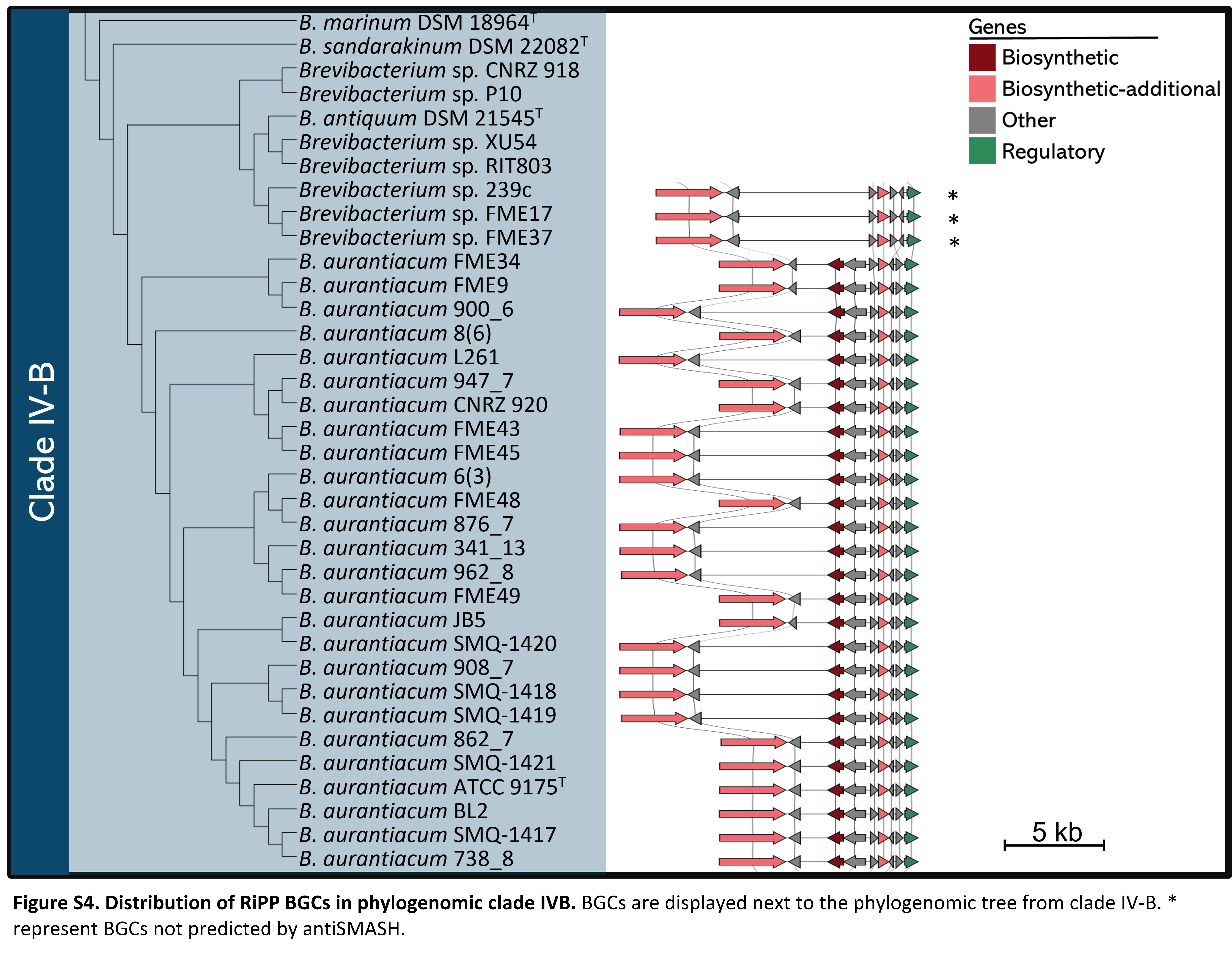

Supplement: Supplementary file 3 — Supplementary Material 3 [file 12864_2023_9694_MOESM3_ESM.png]

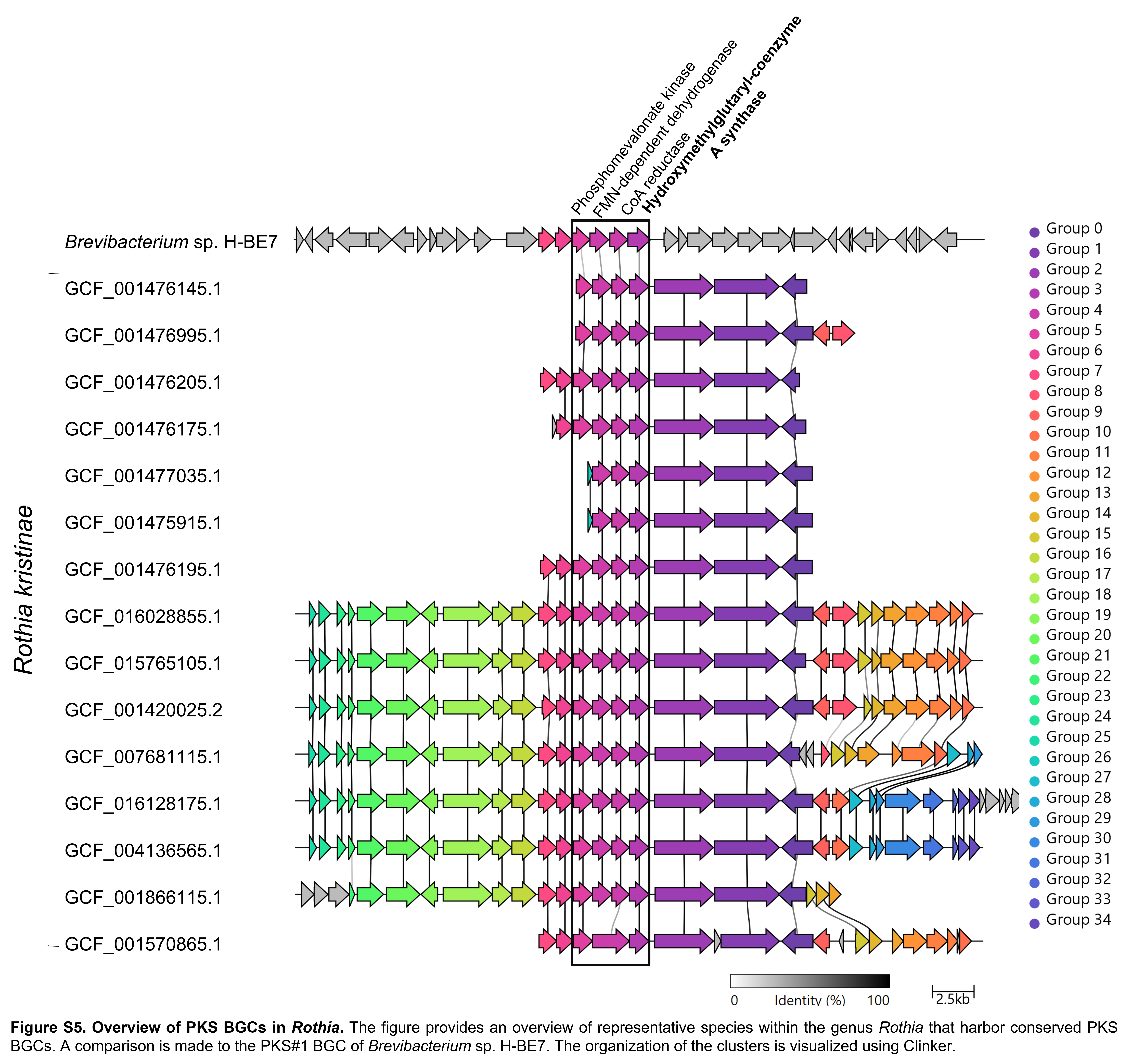

Supplement: Supplementary file 4 — Supplementary Material 4 [file 12864_2023_9694_MOESM4_ESM.png]

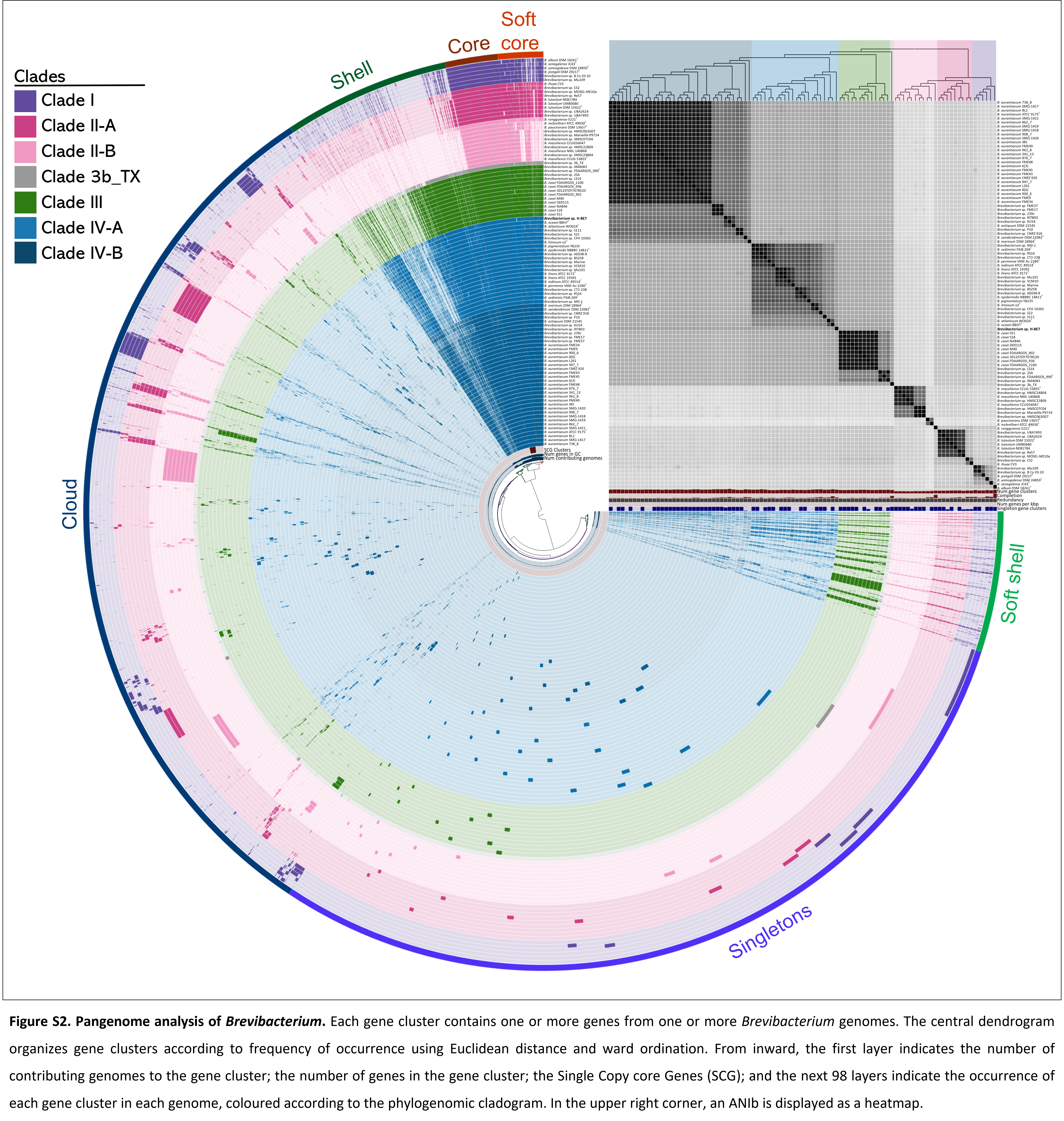

Supplement: Supplementary file 5 — Supplementary Material 5 [file 12864_2023_9694_MOESM5_ESM.png]
